# Acute Appendicitis During Pregnancy: A Case Series of 42 Pregnant Women

**DOI:** 10.7759/cureus.17627

**Published:** 2021-08-31

**Authors:** Tezcan Akın, Birkan Birben, Gökhan Akkurt, Onur Karaca, Mustafa Dönmez, Sadettin Er, Mesut Tez

**Affiliations:** 1 General Surgery, Ankara Bilkent City Hospital, Ankara, TUR; 2 General Surgery, Yildirim Beyazit University Hospital, Ankara, TUR

**Keywords:** pregnancy, acute appendicitis, neutrophil-lymphocyte ratio, platelet-lymphocyte ratio, appendectomy

## Abstract

Introduction

It is difficult to diagnose the symptoms of acute appendicitis in pregnant women due to its similarities with pregnancy physiology. In this study, we examined the diagnostic value of laboratory parameters in the diagnosis of acute appendicitis in pregnant women.

Material and methods

Forty-two patients who underwent appendectomy during pregnancy were evaluated. The demographic characteristics, laboratory parameters and imaging methods of the patients were examined. According to the pathology results, the patients were evaluated in two groups as normal appendix and acute appendicitis. In addition, a non-pregnant control group was formed to compare the results between the pregnant and control groups.

Results

The mean age of the 42 patients was 30±6 years, and the pathology results were evaluated as normal in 16 (38.1%) of the patients. As imaging methods, ultrasonography was undertaken in all patients, with MRI being additionally performed in two patients. When the normal appendix and acute appendicitis groups were compared, no significant difference was observed in terms of laboratory parameters (neutrophil, lymphocyte, white blood cell and platelet counts, neutrophil-lymphocyte ratio, platelet-lymphocyte ratio, mean thrombocyte volume, red cell distribution width, and pregnancy trimesters (P>0.05). The group that had undergone appendectomy had a significantly higher rate of negative appendectomy compared to the control group (P=0.001).

Conclusion

Laboratory parameters alone cannot be sufficient for the diagnosis of acute appendicitis in pregnant patients. If clinical examination, laboratory parameters and USG are not sufficient for diagnosis, MRI is the imaging method that should be considered to reduce negative appendectomy rate.

## Introduction

Acute appendicitis is the most common surgical pathology diagnosed in patients admitted to emergency departments with abdominal pain [1]. Clinically suspected appendicitis is also the most common indication for non-obstetric surgery during pregnancy, with a reported incidence of 1 in 500-2000 pregnancies [2] Acute appendicitis occurs mostly during the second trimester, although it can be seen at any time during pregnancy [3].

Diagnosis of acute appendicitis is often challenging and involves a synthesis of clinical, laboratory, and radiological findings [4]. Pregnant women with acute appendicitis usually apply to gynecology and obstetrics clinics because the causes of pregnancy-related abdominal pain are considered firstly [5]. Accurate and timely diagnosis is important to reduce complication and negative appendectomy rates and generally requires the collaboration of obstetrics and general surgery clinics because delays in diagnosis may cause increased maternal and fetal mortality and morbidity [5]. The use of imaging methods in pregnant patients with appendicitis is limited due to the efficacy of ultrasonography, the ionizing radiation risk of tomography to the fetus, and the lack of widespread use of magnetic resonance imaging [6]. Although the appendix may be difficult to visualize in pregnancy because of anatomical changes, ultrasonography still remains the most commonly used imaging modality to diagnose acute appendicitis [7,8].

Although there is no specific laboratory marker for the diagnosis of appendicitis, various parameters, such as white blood cell (WBC) count, C-reactive protein (CRP), neutrophil-lymphocyte ratio (NLR), and platelet-lymphocyte ratio (PLR) are used to diagnose acute appendicitis [6]. In our study, we examined the diagnostic value of laboratory parameters in the diagnosis of acute appendicitis in pregnant women.

## Materials and methods

Patient data

The approval for the study was obtained from the local ethics committee (approval number: E1-20-1048). Using the database of Ankara City Hospital, 3,132 women aged 18 and over, who underwent appendectomy between January 2015 and July 2020, were retrospectively analyzed. Forty-two patients who underwent appendectomy during pregnancy were included in the study. Open appendectomy was performed in all patients and. Diagnostic or therapeutic laparoscopy was not performed. Laboratory parameters were obtained from blood samples taken at the time of admission to the hospital. The pre-diagnosis of acute appendicitis was made by evaluating the physical examination, laboratory and radiological findings of the patients together. For all patients, the demographic characteristics, neutrophil count, lymphocyte count, white blood cell count, platelet count, NLR, PLR, mean platelet volume (MPV), red cell distribution width (RDW), week of gestation, fetal (abortus) and maternal complications (simultaneous cesarean section), and imaging methods used were evaluated. According to the pathology results, the patients were examined in two groups as normal appendix and acute appendicitis. In addition, the patients with acute appendicitis were evaluated in subgroups as simple and complicated appendicitis (necrosis, gangrenous, and perforated). The control group was randomly formed at one to two ratio with female patients in the same age range, who had undergone open appendectomy but not during pregnancy.

Statistical analysis

Data analysis was carried out using SPPS v. 15 for Windows (IBM Corp, Armonk, USA). The normality tests were performed using the Kolmogorov-Smirnov test. The descriptive statistics of normally distributed continuous variables were given as mean ± standard deviation (SD), and the descriptive statistics of non-normally distributed continuous variables were expressed as median (interquartile range) values. The inter-group differences of normally distributed variables were analyzed with Student’s t-test, while those of non-normally distributed variables were analyzed using the Mann-Whitney U test.

## Results

The mean age of the 42 patients who had undergone appendectomy during pregnancy was 30±6 years. According to the pathology results, 16 (38.1%) patients had a normal appendix. There was no statistically significant difference between the two groups in terms of age, leukocyte count, neutrophil count, lymphocyte count, RDW, MPV, platelet count, NLR, and PLR (P>0.05 for all). While abortus was seen in the first trimester, maternal complications were seen in the third trimester, and there was no significant difference between the groups in terms of complications (P>0.05). Of the 16 patients in the normal appendix group, seven (43.7%) were in the first, five (31.3%) were in the second and four (25%) were in the third trimester. In the acute appendicitis group, 13 (50%) of the 26 patients were in the first trimester, eight (30.8%) in the second trimester, and five (19.2%) in the third trimester. There was no significant difference between the groups in relation to trimesters (P>0.05) (Table 1). It was determined that as the imaging method, ultrasonography had been performed in all patients, and magnetic resonance imaging (MRI) in two patients as an additional imaging modality and the patients who had undergone MRI were in their second or third trimesters.

**Table 1:** Comparison of laboratory parameters, complications, and pregnancy trimesters according to the pathology results in patients that underwent appendectomy during pregnancy RDW, red cell distribution width; MPV, mean platelet volume; NLR; neutrophil-lymphocyte ratio; PLR, platelet- lymphocyte ratio

N(%)	Pregnancy (+)		P value	
	Normal appendix 16 (38.1)	Acute appendicitis 26 (61.9)		
				Age, years	30 ± 6	30 ± 6	0.735	
Leukocytes x10^9^/L	14.03 ± 4.11	14.62 ± 3.18	0.632	
Neutrophils x10^9^/L	11.30 ± 4.30	12.2 ± 3.21	0.469	
Lymphocytes x10^9^/L	1.4 (1.2-2.8)	1.3 (1.1-2.1)	0.260	
RDW %	14 (12.9-14.6)	14.5 (13.8-16.3)	0.428	
MPV fL	8.55 ± 1.38	8.67 ± 1.38	0.808	
Platelets x10^9^/L	273 ± 101	271 ± 56	0.953	
NLR	8.35 (5-9.71)	8.23 (5.13-12.6)	0.301	
PLR	180 ± 102	220 ± 118	0.310	
Abortus	1	1	0.730	
Maternal complications	3	1	0.183	
First trimester of pregnancy	7	13	0.610	
Second trimester of pregnancy	5	8	0.889	
Third trimester of pregnancy	4	5	0.963	

When the patients in the control group were evaluated as those with normal appendix and acute appendicitis according to the pathology results, there was no statistically significant difference in terms of age, leukocyte count, neutrophil count, lymphocyte count, RDW, MPV, platelet count, NLR, and PLR (P>0.05).

When the 42 patients who had undergone appendectomy during pregnancy were compared with the 107 patients in the control group, it was observed that the rate of negative appendectomy during pregnancy was statistically significantly higher in the former (38.1%) than in the latter (13.%) (P=0.001). However, no statistical difference was detected in the rate of complicated appendicitis (P=0.723) (Table 2).

**Table 2:** Comparison of the pregnant women that underwent appendectomy during pregnancy with the controls.

	Pregnancy (+) (n = 42)	Control group (n = 107)	P value
Normal appendix	16 (38.1%)	14 (13.1%)	0.001
Acute appendicitis	26 (54.8%)	93 (81.3%)	0.056
Complicated appendicitis	3 (7.1%)	6 (5.6%)	0.723

## Discussion

Pregnant women are less likely to have a classic presentation of appendicitis, but the most common symptom of appendicitis, such as pain in the lower right quadrant, occurs near McBurney's point in most pregnant women, irrespective of the stage of pregnancy [8]. Diagnosis of acute appendicitis in pregnant women is challenging because symptoms of nausea, vomiting, and abdominal pain can be difficult to discriminate from pregnancy-related symptoms [6]. It is difficult to diagnose acute appendicitis by history and physical examination. Hemogram parameters and imaging methods are used to diagnose however, the use of imaging methods is limited due to possible harmful effects for the fetus and their accessibility. Delay in the diagnosis of acute appendicitis causes increased mortality and morbidity rates for both mother and fetus [9]. Confirmation of early diagnosis in pregnant patients with suspected acute appendicitis is very important. While the diagnosis of acute appendicitis needs to be confirmed urgently, negative appendectomy should also be avoided [3]. Preoperative imaging has led to a significant decrease in the negative appendectomy rate in women and has become a critical part of the diagnostic pathway [7]. Although CT has high accuracy in diagnosing acute appendicitis, the risks of ionizing radiation associated with this imaging technique necessitate consideration of alternative techniques in pregnancy [7]. Ultrasonography and MRI are not associated with ionizing radiation, have not been shown to have any deleterious effects on pregnancy, and should be used when feasible [10]. There are no specific laboratory parameters specific to the diagnosis of acute appendicitis, but WBC and CRP are widely used for this purpose [11]. Even if CRP and WBC are helpful parameters in diagnosing acute appendicitis, it should be considered that they may be high in healthy pregnant women [6] In addition to laboratory parameters like leukocyte count, neutrophil count, lymphocyte count, CRP, RDW, MPV, platelet count, NLR, and PLR, imaging methods are also used for the diagnosis of acute appendicitis in pregnant women [3].

In our study, the laboratory parameters such as WBC, NLR, PLR, MPV, were not found significant for the diagnosis of acute appendicitis in pregnant patients. But Yazar et al. [6] determined that the NLR and PLR levels of pregnant women with acute appendicitis were higher in patients compared to those with normal pregnancy, and Baskıran et al. [12] reported that NLR and PLR were significantly higher in the second and third trimesters of acute appendicitis patients compared to the first trimester. Theilen et al. [13] showed that a white blood cell count value of higher than 18 x109/L was one of the most important parameters for the diagnosis of acute appendicitis in pregnant women, and in another similar study conducted with ​​ pregnant women, Çınar et al. [3] stated that the white blood cell count, neutrophil count, NLR, and PLR were significantly higher in the acute appendicitis group compared to the normal appendix group.

In this study, the imaging method used in all of our patients was ultrasonography, with MRI being additionally undertaken in two patients, and the rate of negative appendectomy was found to be 38.1%. It was observed that the rate of negative appendectomy in our study was high among pregnant patients. In the control group of our study, the rate of negative appendectomy was found to be 13.08%. In the literature, the rate of negative appendectomy differs, being reported as 30% by Arer et al. [14], 14.9% by Çınar et al. [3], and 12.1% by Bazdar et al. [15]. Theilen et al. [13] noted that despite MRI imaging, the rate of negative appendectomy was still high at 33%. Pedrosa et al. [16] also reported that MRI was successful in excluding the diagnosis of acute appendicitis in pregnant women, but its positive predictive value was low. Pedroza et al. [17] determined that the false positivity rate was high and the negative appendectomy rate was 29%. But in a recent meta-analysis, the diagnostic value of MRI in acute appendicitis was reported to be 96%, with a sensitivity and specificity that was either similar or better than CT [18]. In obstetric cases, the rate of negative appendectomy in suspected appendicitis cases was reported as 25-50% in another study [19]. Furthermore, in the current study, the rate of complicated appendicitis in pregnant and non-pregnant women was similar and consistent with the literature [20].

Although negative appendectomy rate in our study is similar to some studies in the literature, it can be qualified as high. We consider that the negative appendectomy rate was high in our sample due to the limited use of MRI in pregnant patients. We think that if MRI had been performed on all patients, the high rate of negative appendectomy could have been reduced.

The limitation of the present study is its retrospective design based on analysis of patient records and a relatively small sample size.

## Conclusions

In conclusion, diagnostic difficulties still continue in pregnant women who are considered to have appendicitis. Laboratory parameters alone are not sufficient for the diagnosis of acute appendicitis in pregnant patients. In cases that cannot be diagnosed with clinical examination, laboratory parameters and USG, if clinical suspicion of acute appendicitis still persists, MRI is the imaging modality that should be considered for diagnosis.
